# Effectiveness of a Health Education Program to Improve Knowledge and Attitude Towards Cervical Cancer and Pap Smear: A Controlled Community Trial in Malaysia

**DOI:** 10.31557/APJCP.2020.21.3.853

**Published:** 2020-03

**Authors:** Rodziah Romli, Sa’adiah Shahabudin, Norkhafizah Saddki, Norehan Mokhtar

**Affiliations:** 1 *Craniofacial and Biomaterials Sciences Cluster, Advanced Medical and Dental Institute, Universiti Sains Malaysia, Bertam, Pulau Pinang, *; 2 *Kolej Pembantu Perubatan Alor Setar, Alor Setar, Kedah, *; 3 *School of Dental Sciences, Universiti Sains Malaysia, Health Campus, Kubang Kerian, Kelantan, Malaysia. *

**Keywords:** Cervical cancer, pap smear, health education, knowledge, attitude

## Abstract

**Objective:**

We examined the effectiveness of a health education program to improve; knowledge and attitude towards cervical cancer and Pap smear, and uptake of Pap smear test among female entrepreneurs in Kedah, a northern state of Malaysia.

**Methods:**

This controlled community trial involved 210 women from the districts of Alor Setar and Sungai Petani. Simple random sampling was applied to select 105 women from each district. Self-administered questionnaires were used to obtain information about the variables of interest. Health education intervention program included educational talk, demo video, experience sharing, pamphlet distribution, and text message reminders. Evaluation of outcomes was performed twice. The text message reminders acted as the cues to action that were sent between the two evaluation times at one-month interval. Women in the control group received educational talk alone. In the control group, evaluation of outcomes was done only once, which was one month after the educational talk.

**Results:**

Knowledge on cervical cancer and Pap smear, and attitude towards Pap smear among women in both intervention and control group improved significantly at Evaluation 1. However, no further improvements were observed in the intervention group at Evaluation. The uptake of Pap smear in the intervention group increased significantly from 48.0% at Baseline to 68.0% at Evaluation 1 (P<0.001), and from 68.0% to 79.0% at Evaluation (P<0.001). A significant increase in Pap smear uptake was also seen in the control group from 63.0% at Baseline to 76.0% at Evaluation stage 1 (P=0.003).

**Conclusions:**

Educational talk alone was effective in improving knowledge on cervical cancer and Pap smear, attitude towards the test, and the actual uptake of the test. However, text reminders were more effective than having an educational talk alone in increasing uptake of Pap smear test among participants.

## Introduction

Cervical cancer is the fourth most common cancer in women worldwide (World Health Organization, 2018). In 2012, 528,000 new cases of cervical cancer were diagnosed, and in the same year, 266,000 women around the world died of cervical cancer with most deaths occurred in low- and middle-income countries (World Health Organization, 2014). In Malaysia, Cervical cancer rank seventh among all cancer with frequency of 4.2% and rank third among women with frequency of 7.7%. (Azizah et al., 2016). The National Cancer Registry report also showed that most cervical cancer patients presented at stage II (35.8%), followed by stage I (23.9%), stage III (21.9%), and stage IV (18.4%) (Azizah et al., 2016).

Cervical cancer is preventable through screening tests to detect early changes in cervical cells or tissues, and Papanicolaou (Pap) smear test is possibly the most widely used cervical cancer screening test (Safaeian et al., 2007). Pap smear test has been successful in reducing the incidence and mortality from cervical cancer, particularly in high-income countries with a decrease in incidence rate by as much as 80% since it was first introduced in the 1940s (Sankaranarayanan, 2014). Screening programs offering Pap smear test however were less successful in low- and middle-income countries mainly due to lack of infrastructure and resources including technical, medical, and financial, and a lack of awareness about cervical cancer among women and health-care providers (Sankaranarayanan, 2014).

The Pap smear test is the primary cervical cancer screening method in Malaysia and it is available free of charge at primary health care facilities throughout the country. Screening is recommended to all women between the ages of 20 to 65 years who are, or have been sexually active (Ministry of Health Malaysia, 2003). The test is done for two consecutive years and if both results are negative, re-screening of every three to five years is recommended (Ministry of Health Malaysia, 2003). Despite the free services, the uptake of Pap smear test among women in Malaysia has been shown to be low (Al-Naggar et al., 2010; Abdullah et al., 2011; Gan and Dahlui, 2013).

In-depth interviews conducted on 20 Malaysian women who have never had a Pap smear test showed that the women lacked the knowledge about cervical cancer screening using Pap smear test including the indications and benefits, and their perception to cervical cancer susceptibility was low (Wong et al., 2008; Wong et al., 2009). Other barriers included embarrassment, fear of pain, misconceptions about cervical cancer, fatalistic attitude, and undervaluation of their own health and family needs (Wong et al., 2008). In addition, preoccupation with work appeared to be the main reason for not doing the test among highly educated working women (Baharom and Ismail, 2008; Abdullah et al., 2013). The increasing trend of female labour force participation rate in Malaysia from 42.2% in 1980 to 46.7% in 2000, and to 54.7% in 2017 (Ministry of Women and Family Development Malaysia, 2003; Department of Statistics Malaysia, 2018) highlighted the need for an intervention program targeted at working women to deliver accurate information about cervical cancer as well as the purpose and importance of Pap smear screening. 

A controlled community trial was undertaken to examine the effectiveness of a health education program to improve knowledge and attitude towards cervical cancer and Pap smear test, as well as the uptake of Pap smear test among female entrepreneurs in Kedah, a northern state of Malaysia.

## Materials and Methods

*Study Population and Sample*

Female entrepreneurs in the state of Kedah who received financial help from Amanah Ikhtiar Malaysia (AIM), were chosen to represent a sample of working women. AIM is the largest microcredit organization in Malaysia that aims to alleviate poverty by supporting entrepreneurship through the provision of interest free microcredit facilities to the poor and low-income households to finance income-generating activities. The recipients of AIM funding, known as Sahabat AIM, who were married, or previously married were eligible to participate in this study. We also required the women to have a mobile phone, with or without internet coverage, because our health education program use short message service (SMS) texts to remind the women about having Pap smear test done if they have not done so. Women with a history of cervical cancer or have been diagnosed with cervical cancer were excluded to avoid bias. The ethical approval to conduct this study was obtained from the Universiti Sains Malaysia Human Research Ethics Committee [USM/JEPeM/16010010]. Additionally, permission from the AIM was also obtained [AIM/ UPI/800-040/01]. The districts of Alor Setar and Sungai Petani were chosen as the study area due to similarities in social demography, geography, and availability of healthcare facilities and services. 

The districts were randomly assigned as the intervention group and control group. Alor Setar district was assigned as the intervention group and Sungai Petani as the control group. Sample size was calculated using the Power and Sample Size Calculations software version 3.1.2. for comparing two means. With an expected difference in mean scores of 2, a standard deviation of 4.9 (Frenkel et al., 2002), type II error of 80%, and type I error of 5%, a sample size of 95 subject for each group was estimated. In anticipation of 10% non-response rate, a sample size of 105 women in each intervention and control group was decided.

There were about 1,500 Sahabat AIM in the district of Alor Setar and 1,200 Sahabat AIM in the district of Sungai Petani. Each district has 30 meeting zones with 30 to 60 Sahabat AIM in each zone. Simple random sampling was used to select 3 meeting zones per district prior to the study, and the names of Sahabat AIM from all 6 meeting zones were obtained. The women were screened for eligibility, and simple random sampling was applied to select 35 women from each zone to get the prior determined sample size of 105 women from each district.

*Research Tools*

The women’s knowledge about cervical cancer and Pap smear test was assessed using a 30-item self administered questionnaire. The questions represented two domains of knowledge: knowledge on symptoms, risk factors and treatment options for cervical cancer (18 items) and knowledge on purpose and procedures of Pap smear test, the recommended group for screening, the frequency and the best time for screening (12 items). The items were taken from a questionnaire developed by researchers from Universiti Sains Malaysia, Universiti Kebangsaan Malaysia and Universiti Malaya for the Household Survey on Cancer of Cervix and Pap Smear Screening under the Intensified Research in Priority Areas (IRPA) grant (Grant no: 06-02-1032 PR24/09-06). The original questionnaire was in Bahasa Malaysia. The response options given were: “True”, “False” and “Don’t know”. Each correct answer receives 1 mark, and incorrect answers and “Don’t know” responses do not receive any mark. The total score for knowledge on cervical cancer and Pap smear may range between 0 to 18 and 0 to 12 respectively.

A questionnaire by Abdullah et al., (2011) was used to assess the women’s attitude towards Pap smear. The self-administered questionnaire, also originally in Bahasa Malaysia, has 17 items that were all negatively worded, measured using a 5-point Likert scale: 5 for ‘strongly agree’, 4 for ‘agree’, 3 for ‘not sure’, 2 for ‘disagree’ and 1 for ‘strongly disagree’. The scores may range between 17 and 85, with a lower score indicating more positive attitude. 

Before being used, the questionnaires were tested for internal consistency among 50 Sahabat AIM in Pendang, another district in the state of Kedah. Based on similar inclusion and exclusion criteria, simple random sampling was done to select participants from the name list of female entrepreneurs registered with the Pendang branch. The Cronbach’s alpha for the entire set of questionnaires was 0.753, and the values for each domain are as follows: 0.849 for knowledge on cervical cancer, 0.660 for knowledge on Pap smear, and 0.844 for attitude towards Pap smear screening. 

Additionally, a structured form was used to ask about the women’s experience of having Pap smear test and to collect information on selected characteristics, namely: age, ethnic group, highest education level, personal monthly income, marital status, pregnancy experience, family planning method used, and menopausal status. 

A standardized health education program was designed to include the following: 1) A 30-minute educational talk on cervical cancer and Pap smear test. The talk that explains about stages, symptoms, risk factors and treatment for cervical cancer, the importance of Pap smear and its procedures, was part of the conventional health education services provided by the Ministry of Health Malaysia (MOH). With an aid of PowerPoint slides, the talk was delivered by a MOH Obstetrics and Gynaecology specialist; 2) A 5-minute video on Pap smear test procedures. The video was a narrative motion graphic video titled “A cervical screening test – What can you expect” by Sanne Stravens, accessible for public use on the YouTube channel. The original language was English and it was dubbed into Malay for use in this study; 3) Experience sharing from a cervical cancer survivor. The invited survivor shared her experience of having Pap smear test, being diagnosed with cervical cancer, undergoing the treatment, and facing the impact of the disease and treatment. The session was moderated by the main author; 4) Distribution of pamphlet on cervical cancer and Pap smear test. The pamphlet was produced by the MOH; and 5) SMS text reminders. Two text reminders were sent to the women over a 3-month period. The first reminder highlighted Pap smear test as a cervical cancer screening method, and the second reminder was a caption “Lets us do Pap smear screening”.

*Data Collection*

Eligible women who were selected and agreed to participate were approached after their weekly zone meeting at their respective meeting zones. Written informed consent was obtained prior to data collection. Baseline information on knowledge, attitude and practice was first obtained from the women in both intervention and control group.

*Intervention Group*

Following initial questionnaire administration, the health education intervention program on cervical cancer and Pap smear was delivered to the women using the materials and methods previously explained. The face-to-face health education lasted for about an hour. Reassessment of the women’s knowledge, attitude and practice was done twice. The first evaluation (Evaluation 1) was done one month after the face-to face intervention, and the second evaluation (Evaluation 2) was done three months after Evaluation 1. SMS text reminders were sent between the two evaluation time, at one-month interval. Women in the control group received the MOH 30-minute educational talk on cervical cancer and Pap smear test by the same specialist who delivered the talk for the intervention group. The PowerPoint presentation used was also the same as used for the intervention group. Re-evaluation of knowledge, attitude and practice of women in the control group was done one month after the educational talk (Evaluation 1).

*Data Collection*

Data were processed and analysed using IBM SPSS Statistics v22.0. Descriptive statistics of the variables were determined: frequency and percentage for categorical data, and mean and standard deviation for numerical data. A chi-square test was used to compare the characteristics of women in the intervention and control group. An independent t-test was used to compare mean cervical cancer knowledge scores, mean Pap smear knowledge scores and mean Pap smear attitude scores between women in intervention and control group. The effect of time on mean scores changes in the intervention group was assessed using repeated measure ANOVA. Pap smear uptake at Baseline, Evaluation 1, and Evaluation 2 were compared using a McNemar test. 

## Results

*Characteristics of participants*

Table 1 shows characteristics of women in the intervention and control group, which were mainly similar. Most women were between 35 and 49 years old, with a mean age of 41 years (SD 10.03) and 44 years old (SD 10.46) in the intervention and control group, respectively. The predominant ethnic group was Malay. Most women completed their education up to secondary school and the highest personal monthly income was MYR1,000. Most women were married and were still in the reproductive age group. About half of women in the intervention group had done Pap smear (48.0%) while more than half of women in the control group (63.0%) have had the test done, giving an overall percentage of 55.2%.

*Comparison of mean cervical cancer and Pap smear knowledge scores, and mean Pap smear attitude scores between women in intervention and control group*

The number of participants in both groups reduced from 105 at Baseline to 101 at Evaluation 1. Table 2 shows the mean cervical cancer knowledge scores, mean Pap smear screening knowledge scores, and mean Pap smear attitude scores in intervention and study groups at Baseline and Evaluation 1. No significant difference was seen in all mean scores between the groups.

*The effect of time on mean cervical cancer knowledge scores, mean Pap smear knowledge scores and mean Pap smear attitude scores in intervention group*

At Evaluation 2, the number of participants in the intervention group further reduced to 96 from 101. Table 3 shows results of repeated measures ANOVA with Greenhouse-Geisser correction on the changes in mean cervical cancer knowledge scores, Pap smear knowledge scores, and Pap smear attitude scores in the intervention group across time. All residuals were approximately normally distributed. 

Our results showed that the mean cervical cancer knowledge scores differed significantly between time points [F(1.51,142.96)=146.66, P<0.001]. Post hoc tests using the Bonferroni correction revealed that the mean knowledge score increased significantly between Baseline and Evaluation 1. No significant increase in mean cervical cancer knowledge score was observed after SMS reminders between Evaluation 1 and Evaluation 2.

Similarly, the mean Pap smear knowledge scores and mean Pap smear attitude scores differed significantly across time, [F(1.76,167.23)=54.53, P<0.001] and [F(1.69,161.16)=49.66, P<0.001] respectively. Post hoc tests using the Bonferroni correction revealed that knowledge and attitude scores differed significantly between Baseline and Evaluation 1, but no significant changes in mean scores were observed after SMS reminders between Evaluation 1 and Evaluation 2.

*The effect of time on mean cervical cancer knowledge scores, mean Pap smear knowledge scores and mean Pap smear attitude scores in control group*

Table 4 shows results of comparison of mean cervical cancer knowledge scores, mean Pap smear knowledge scores, and mean Pap smear attitude scores among participants in the control group between Baseline and Evaluation 1. Changes in scores between Baseline and Evaluation 1 were all significant (P<0.001).

*Comparison of Pap smear uptake among women in intervention and control group across time*

Changes in the proportion of participants who had Pap smear test done at different evaluation times are shown in Table 5. The uptake of Pap smear in the intervention group increased significantly from 48.0% at Baseline to 68.0% at Evaluation 1 (P<0.001), and from 68.0% to 79.0% at Evaluation 2 (P<0.001). A significant increase in Pap smear uptake was also seen in the control group from 63.0% at Baseline to 76.0% at Evaluation 1 (P=0.003).

**Table 1:** Based Line Characteristic of Women in Intervention Group and Control Group

Variable	Frequency (%)		P value^a^
	Intervention group	Control group	
	(n=105)	(n=105)	
Age group (year)			
20 – 34	30 (28.6)	24 (22.9)	0.322
35 – 49	46 (43.8)	43 (41.0)	
50 – 65	29 (27.6)	38 (36.2)	
Ethnic group			
Malay	102 (97.1)	105 (100.0)	0.218
Indian	1 (1.0)	0 (0.0)	
Siamese	2 (1.9)	0 (0.0)	
Highest educational level			
No formal qualification	1 (1.0)	3 (2.9)	0.168
Primary school	7 (6.7)	16 (15.2)	
Lower Secondary school	24 (22.9)	27 (25.7)	
Upper Secondary school	59 (56.2)	52 (49.5)	
Certificate	5 (4.8)	1 (1.0)	
Diploma/ vocational	8 (7.6)	6 (5.7)	
Bachelor/Master/PhD	1 (1.0)	0 (0.0)	
Personal monthly income (MYR)			
≤ 1,000	59 (56.2)	64 (61.0)	0.631
1,001 – 3,000	44 (41.9)	38 (36.2)	
≥ 3,001	2 (1.9)	3 (2.9)	
Marital status			
Married	91 (86.7)	88 (83.8)	0.136
Separated	2 (1.9)	1 (1.0)	
Divorced	7 (6.7)	3 (2.9)	
Widowed	5 (4.8)	13 (12.4)	
Pregnancy experience			
Yes	88 (83.8)	90 (85.7)	0.514
No	17 (16.2)	15 (14.3)	
Family planning method			
None	77 (73.3)	67 (63.8)	0.23
Hormonal (pill/injection)	21 (20.0)	30 (28.6)	
Intrauterine device	5 (4.8)	5 (4.8)	
Condom	2 (1.9)	3 (2.9)	
Menopause			
Yes	17 (16.2)	29 (27.6)	0.045
No	88 (83.8)	76 (72.4)	

^a^, Chi-Square test

**Table 2:** Differences in Mean Cervical Cancer Knowledge Scores, Mean Pap Smear Knowledge Scores, and Mean Pap Smear Attitude Scores between Intervention and Control Group

	Mean (SD)		t-statistics	
Variable	Intervention group	Control group	(d.f.)^a^	P value^a^
Baseline				
Knowledge of cervical cancer	6.0 (3.90)	6.2 (3.80)	0.30 (208.00)	0.734
Knowledge of Pap smear	6.7 (2.54)	6.8 (2.85)	0.05 (208.00)	0.959
Attitude towards Pap smear	47.0 (10.12)	45.4 (8.59)	-1.22 (202.64)	0.224
Phase 1				
Knowledge of cervical cancer	11.5 (2.79)	10.8 (3.94)	-1.33 (180.02)	0.187
Knowledge of Pap smear	9.0 (1.89)	9.1 (2.03)	0.14 (199.00)	0.887
Attitude towards Pap smear	38.5 (10.48)	40.9 (10.72)	1.55 (199.00)	0.124

^a^, Independent t-test

**Table 3:** Differences in Mean Cervical Cancer Knowledge Scores, Mean Pap Smear Knowledge Scores and Mean Pap Smear Attitude Scores Across Time in Intervention Group

Variable	Mean (95% CI)	F-statistics (d.f.)^a^	P value^a^
Knowledge of Cervical cancer			
Baseline	6.0 (5.49, 7.04)	146.66 (1.51, 142.96)	<0.001
Phase 1	11.5 (10.91, 12.05)		
Phase 2	11.8 (11.18, 12.39)		
Knowledge of Pap smear			
Baseline	6.7 (6.43, 7.42)	54.53 (1.76, 167.22)	<0.001
Phase 1	9.0 (8.62, 9.40)		
Phase 2	9.2 (8.86, 9.52)		
Attitude towards Pap smear screening			
Baseline	47.0 (44.28, 48.32)	49.66 (1.69, 161.16)	<0.001
Phase 1	38.5 (36.46, 40.75)		
Phase 2	37.1 (34.95, 39.16)		

^a^, Repeated measures ANOVA

**Table 4:** Differences in Mean Cervical Cancer Knowledge Scores, Mean Pap Smear Knowledge Scores and Mean Pap Smear Attitude Scores Across Time in Control Group

Variable	Mean difference (95% CI)	t-statistics (d.f.)^a^	P value^a^
Knowledge of Cervical cancer			
Baseline-phase 1	4.6 (3.95, 5.53)	11.84 (100)	<0.001
Knowledge of Pap smear			
Baseline-phase 1	2.3 (1.72, 2.93)	7.65 (100)	<0.001
Attitude towards Pap smear screening			
Baseline-phase 1	-4.50 (-6.19, -2.82)	-5.31 (100)	<0.001

^a,^ Paired t-test

**Table 5:** Comparison of Pap Smear Uptake between Women in Intervention and Control Group Across Time

Variable	Pap smear uptake (%)	P value^a^
Intervention group		
Baseline	48	<0.001
Evaluation 1	68	
Baseline	48	<0.001
Evaluation 2	79	
Evaluation 1	68	0.004
Evaluation 2	79	
Control group		
Baseline	63.0	0.003
Evaluation 1	76.0	

^a^, McNemar test

## Discussion

This study investigated the effectiveness of a health education program to improve knowledge and attitude towards cervical cancer and Pap smear test and uptake of the test in a sample of female entrepreneurs in Malaysia. Employing the Health Belief Model to explain and predict Pap smear uptake among the participants (Rosenstock, 1966), our health education program was designed to tackle the four constructs representing the perceived threat and benefits, namely perceived susceptibility, perceived severity, perceived benefits, and perceived barriers through the educational talk, demo video, and experience sharing. Besides the perceived threats and benefits, the Health Belief Model theorizes that a cue, or trigger, is necessary for prompting engagement in health-promoting actions (Rosenstock, 1966). In this study, pamphlet and the SMS reminders served as the cues or triggers that would activate readiness to change and stimulate the desired behaviours. The intervention group received the health education program while the control group received only educational talk. The level of knowledge about cervical cancer and Pap smear, and attitude towards Pap smear test between the interventionand control group at baseline were comparable. 

Educational talk has been shown to be a successful method to increase knowledge of participants regarding cervical cancer and Pap smear (Wright et al., 2011; Adamu et al., 2012; Al Thani et al., 2012; Rosser et al., 2015). In agreement, while significant improvement was found in the level of knowledge and attitudes of women in the intervention group following educational talk, video show, and experience sharing, a significant improvement was also observed in level of knowledge and attitudes of women in the control group who received educational talk alone. Additionally, the mean knowledge and attitudes scores between women in the intervention group and control group following respective input were not significantly different, indicating very small contribution of demo video and experience sharing to improve knowledge and attitudes of the women. 

Pap smear screening is a widely used method for detection of cervical cancer in women with sensitivity and specificity of 55.4% and 96.8% respectively (Mayrand et al., 2007). Despite the test being offered for free at primary care facilities in Malaysia, the uptake of Pap smear test in this country has been low. The rate of uptake ranged from 6.0% among university students to 38.0% among secondary school teachers, and 48.9% among rural women from the state of Perak (Al-Naggar et al., 2010; Abdullah et al., 2011; Gan and Dahlui, 2013). The very low prevalence of Pap smear uptake among the university students is reasonable given that most respondents (74.0%) were single (Al-Naggar et al., 2010). In this study, 55.2% of our participants have had a Pap smear test done. Our participants were selfemployed and the highest education level for most was secondary school. The prevalence of Pap smear uptake in our study is comparable with that reported among rural women in Perak who mostly attained only secondary education and were unemployed (Gan and Dahlui, 2013), but higher than the secondary school teachers who were mostly university graduates (Abdullah et al., 2011). These findings corroborate results of previous studies that working commitment was a significant barrier toward having a Pap smear test among highly educated working women (Baharom and Ismail, 2008; Abdullah et al., 2013).

In this study, measurement of outcomes in the intervention group was done twice. This is in line with the observation that health beliefs and behaviours may change over time, rendering a one-time outcome measurement invalid (Carpenter, 2010). Cues to action may occur after the first measurement, and if the trigger is strong enough, adoption of prevention behaviour may transpire despite initial low perceptions to health threats and benefits (Rosenstock, 1966). Our cues to action were given at two different time points; the pamphlet was given immediately following the educational talk, demo video, and experience sharing before the first evaluation, and the SMS text reminders were delivered after the first evaluation.

While the SMS text reminders failed to improve the knowledge and attitudes of women in the intervention group further, the reminders had successfully promoted a continued increase in the uptake of Pap smear test by another 11.0% after the initial increase of 20.0% following the educational talk, demo video, experience sharing, and distribution of pamphlet. Nevertheless, the time interval between the second SMS reminder and the evaluation was relatively short at 1 month, which may limit the likelihood for the cue to take effect. Participants may have had the intention to undergo screening, but have not found a suitable time to take the test.

The influence of time on health-related behaviour was demonstrated in a study among Korean American women in the United States; the positive effect of mobile phone text messaging on participants’ intention to receive Pap test was evident, but the increase was not statistically significant due to a very short interval that was not sufficient to bring about the behaviour change (Lee et al., 2014). The authors also found a significant decrease in perceived socio-cultural barriers to cervical cancer screening at 3-month follow up, and postulated that longer follow up period may be more effective for those with more barriers (Lee et al., 2014). Additionally, although previous studies have shown that single educational talk failed to improve Pap smear screening practice despite an increase in knowledge (Wright et al., 2011; Adamu et al., 2012), our study has proved that single educational talk could significantly increase the uptake of Pap smear test, although the increment (13.0%) was lower than that observed in the intervention group.

In conclusion, educational talk alone was effective in improving knowledge of participants regarding cervical cancer and Pap smear, attitude towards the test, and the actual uptake of the test. Although SMS text reminders failed to improve the knowledge and attitude of those in the intervention group further, the impact of reminders on Pap smear uptake was very encouraging and superior than having educational talk alone.

## Funding

This study was funded by the Universiti Sains Malaysia short term grant (304/CIPPT/6313301).
